# How many suspected adult ADHD cases actually receive the diagnosis? Related factors in an assessment-seeking sample

**DOI:** 10.1017/S1092852926101175

**Published:** 2026-09-22

**Authors:** Gianluca Rosso, Camilla Perotti, Beatrice Defilippi, Camilla Garrone, Giuseppe Maina, Gabriele Di Salvo

**Affiliations:** 1 Neuroscience “Rita Levi Montalcini”, University of Turin, Italy; 2 San Luigi Gonzaga University Hospital, Italy

**Keywords:** Attention-deficit hyperactivity disorder, predictive factors, differential diagnosis, adult assessment, comorbidities, overdiagnosis

## Abstract

**Objective:**

Adult ADHD assessments have increased sharply in recent years, yet the diagnostic yield and predictors of confirmed ADHD among assessment-seeking individuals remain insufficiently understood. This study aimed to examine the sociodemographic and clinical characteristics of adults self-referred or referred by clinicians for suspected ADHD, to determine confirmation rates, factors associated with confirmation, and alternative diagnoses.

**Methods:**

A cross-sectional study was conducted on 232 adults evaluated at a regional ADHD referral center. Assessments included semi-structured clinical interviews and standardized instruments (DIVA, SCID-5, ADHD-RS). Logistic regression was used to examine factors associated with the final clinical ADHD diagnosis.

**Results:**

Overall, 64.2% of individuals received an ADHD diagnosis. Compared with non-ADHD participants, those diagnosed with ADHD were more frequently referred by other healthcare professionals and displayed greater academic impairment. They also showed higher rates of mood swings and delayed sleep onset. Childhood inattention emerged as the only DIVA interview score significantly associated with an ADHD diagnosis. Among individuals without ADHD, the most frequent alternative diagnoses were anxiety disorders and depressive disorders, followed by personality disorders, substance use disorders, and bipolar disorders.

**Conclusions:**

Adult ADHD emerges as a multifactorial condition shaped by clinical and functional factors. The final clinical diagnosis was most strongly associated with structured clinical tools, functional impairments (in the academic domain), emotional-regulation difficulties, and childhood inattention, whereas self-reported and more general factors were less informative. These findings underscore the importance of a developmentally informed and comprehensive assessment approach, given the high prevalence of psychiatric conditions that may mimic ADHD.

## Introduction

Attention-deficit/hyperactivity disorder (ADHD) is a neurodevelopmental disorder characterized by persistent patterns of inattention, hyperactivity, and impulsivity, with symptoms emerging in childhood and often persisting into adulthood.^1^ ^,^^2^ The clinical manifestation in adults typically shifts toward inattention, executive dysfunction, and emotional dysregulation, while overt hyperactivity may diminish and present as internal restlessness.^3^ Diagnosis in adults is challenging due to the need for retrospective assessment of childhood symptoms, the developmental instability of symptom clusters, and frequent psychiatric comorbidities, particularly anxiety and mood disorders.^2^ ^,^^4^

In recent years, requests for ADHD assessments in adulthood have risen dramatically worldwide. For instance, in the United States, a national survey reported that 25% of adults suspect they may have undiagnosed ADHD.^5^ Similarly, in Australia, the number of ADHD prescriptions has more than doubled in the past five years, reflecting a sharp increase in both recognition and diagnostic demand. Analysis of data from the Italian Methylphenidate Registry between 2019 and 2025 showed an exponential increase in newly treated patients with ADHD, both in pediatric and adult populations, which is expected to further increase in 2025.^6^ This global trend has been amplified since 2019 by pandemic-related lockdowns, the influence of social media platforms such as TikTok—where ADHD has garnered over 36 billion views—and growing media attention. However, the literature has shown that more than half of the online content related to ADHD on TikTok is misleading.^7^

Therefore, this surge in self-identification is a double-edged sword. While increased awareness has promoted help-seeking among previously overlooked groups, there is also growing concern about potential overdiagnosis and diagnostic inaccuracy. Many individuals presenting for ADHD evaluation base their suspicions on self-report questionnaires, which are often nonspecific and prone to false positives.^8^ Indeed, self-report scales such as the CAARS frequently fail to distinguish ADHD symptoms from those of other psychiatric conditions, particularly anxiety, depression, bipolar disorder, and PTSD.^9^ ^,^^10^ ^,^^11^ False positive rates ranging from 20% to 67% have been reported,^12^ ^,^^13^ whereas other studies highlight possible underreporting of symptoms among true ADHD cases.^14^

Diagnostic complexity is further heightened by the overlap of ADHD symptoms with other clinical presentations and by possible motivational factors. Indeed, potentially every symptom of adult ADHD, including inattention, impulsivity, restlessness, organizational difficulties, and emotional instability, is nonspecific and is also observed in mood disorders, anxiety, personality disorders, substance use disorders, and medical conditions such as sleep or thyroid disorders.^11^ ^,^^15^ Furthermore, some adults may seek an ADHD diagnosis for secondary gains—such as access to stimulant medication or workplace accommodations—raising concerns about symptom exaggeration.^16^ ^,^^17^ Consequently, clinical guidelines recommend incorporating collateral information from external sources (e.g., family members) to strengthen the diagnostic process.^18^

Although the number of adults seeking ADHD assessment has more than doubled in recent years,^19^ the actual diagnostic yield and sociodemographic predictors within this assessment-seeking population remain poorly understood. Recent work in referral samples has shown that up to one-third of individuals evaluated for suspected ADHD ultimately receive a different diagnosis, most commonly anxiety or mood disorders.^8^ ^,^^20^ While some studies have explored diagnostic rates and related aspects such as quality of life,^21^ to our knowledge, there is only a single study—conducted in Iceland—that has systematically characterized who these individuals are, what leads them to seek an ADHD evaluation, and which factors are associated with receiving a confirmed diagnosis.^22^ By contrast, most of the available evidence is solely based on samples “pre-selected” through prior evaluations by specialists, rather than on individuals who independently sought an assessment for suspected ADHD. Eventually, the limited research available has primarily focused on children,^23^ while very few studies have specifically examined these aspects in adults.

Given these gaps, the present study aimed to:

- Analyze the sociodemographic and clinical characteristics of adults referred to a specialized center for suspected ADHD;
- Identify the percentage of confirmed ADHD diagnoses and the sociodemographic or clinical factors associated with diagnostic confirmation;
- Examine alternative diagnoses among those who sought ADHD assessment but did not meet diagnostic criteria.

To our knowledge, this is the first Italian study examining diagnostic confirmation in an adult ADHD referral sample using a multidomain clinical framework including DIVA-based assessment, functional impairment, emotional dysregulation, and sleep-related variables.

## Materials and methods

### Study design and sample

This is a cross-sectional observational study.

All patients who visited the regional reference center for adult ADHD at the Psychiatry Unit of San Luigi Gonzaga University Hospital (Orbassano, Turin) with a request for an ADHD evaluation—whether self-referred or referred by other mental health professionals—were included in the study.

Potential participants were thoroughly explained aims and study procedures, and they had to give their written consent before participating; exclusion criteria included age < 18 and refusal to consent participating in the study. No patients met the exclusion criteria. The protocol was approved by the local Ethical Committees (number 196/2024).

### Assessment

For all the patients, data were collected by an experienced psychiatrist through a semi-structured interview, conducted over three to four sessions, depending on the complexity of the clinical case. The interview covered the following areas:

a) Sociodemographic data: age, sex, marital status, educational level, and occupational status.

b) General clinical assessment: current symptoms, areas of functional impairment, family history of psychiatric disorders, and medical comorbidities. Diagnoses were established based on clinical evaluation and the Italian version of the *Structured Clinical Interview for DSM-5 Axis I Disorders* (SCID-5), a standardized semi-structured clinician-administered interview for the assessment of DSM-5 psychiatric disorders.^24^

c) Exploration of ADHD symptomatology: presence and severity of symptoms during childhood and adulthood were assessed using the *Diagnostic Interview for ADHD in Adults* (DIVA),^25^ a semi-structured clinician-administered interview based on DSM-5 criteria that evaluates the 18 core ADHD symptoms (9 items evaluating inattention, 9 items evaluating hyperactivity/impulsivity) across childhood and adulthood, along with associated functional impairments, with input from a caregiver regarding childhood symptoms. When a caregiver was not available, symptomatology was assessed using the Barkley Adult ADHD Rating Scale–IV (BAARS-IV),^26^ a self-report scale assessing current and retrospective childhood ADHD symptoms aligned with DSM criteria, including inattention, hyperactivity, impulsivity, and sluggish cognitive; current symptom levels were measured clinically and through the *ADHD Rating Scale IV* (ADHD-RS IV),^27^ an 18-item self-report scale reflecting DSM symptoms of inattention and hyperactivity/impulsivity (9-items), to capture the individual’s subjective perception of symptom severity. Additional ADHD-related symptoms—including mood swings, anger outbursts, low self-esteem, low frustration tolerance, and delayed sleep onset—were also examined. Delayed Sleep onset was operationalized during the clinical interview based on the patient’s subjective report, and defined as a prolonged delay in the initiation of sleep, characterized by difficulty falling asleep within a conventional or desired time period after going to bed. Mood swings were assessed during the clinical interview as the presence of rapid and frequent fluctuations in emotional state (e.g., shifts from irritability to low mood) reported by the patient and judged clinically significant by the evaluating psychiatrist. These fluctuations were distinguished from episodic mood changes characteristic of bipolar disorder based on their short duration, high reactivity to environmental stimuli, and lack of sustained mood episodes, consistent with previous literature describing emotional lability in ADHD as characterized by volatile and rapidly changing emotional states.^4^ ^,^^28^

The final diagnosis of ADHD was established clinically by an experienced psychiatrist according to DSM-5 criteria, based on a comprehensive assessment integrating multiple sources of information, including the DIVA interview, clinical history, access to report cards, functional impairment across domains, and the exclusion of alternative explanations. The DIVA was used as a structured assessment tool to support clinical evaluation, but not as a standalone diagnostic instrument. For participants who received an ADHD diagnosis, the clinical subtype of ADHD, age at diagnosis, and age at first ADHD treatment were recorded.

### Statistical analysis

No a priori power calculation was performed, as the study included a consecutive series of patients referred for ADHD assessment during the study period.

The sociodemographic and clinical features of the patients were summarized as mean and SD for continuous variables and as frequency and percentage for categorical variables. We tested the distribution of continuous variables with the Kolmogorov–Smirnov test.

The sample was divided in the following subgroups: patients diagnosed with ADHD (ADHD group) versus patients not diagnosed with ADHD (non-ADHD group).

Because the distribution was not normal (*p* < 0.001), comparisons were performed using χ^2^ tests for categorical variables and the Kruskal–Wallis test for continuous variables.

Binary logistic regression was then used to identify explanatory variables associated with ADHD diagnosis, considering ADHD diagnosis as the dependent variable. Only variables that were statistically significant in the univariate analysis were entered into the multivariable logistic regression model. Cases with missing data on one or more variables included in the regression model were excluded using a complete-case (listwise deletion) approach. Specifically, missing data were related to the absence of DIVA assessment, missing educational level, or non-evaluable occupational functioning, resulting in a final regression sample of 193 participants. A probability of 0.05 was required for inclusion in the equation. The group comparison results were presented as two-sided *p*-values rounded to three decimal places. The criterion for statistical significance in all comparisons was a *p*-value <0.05.

Multicollinearity among predictor variables was assessed using variance inflation factors (VIF) and tolerance values.

All statistical analyses were performed by SPSS software version 29.0.1.0.

## Results

A total of 232 patients attended their first evaluation for suspected adult ADHD. The sample’s demographic and clinical characteristics are summarized in Table 1.

**Table 1.** Sociodemographic and Clinical Characteristics of the Sample Table 1. long description.

Characteristics	Sample (*n* = 232)
Sex, *n* (%)	
Male	134 (57.8%)
Female	98 (42.2%)
Marital status, *n* (%)	
Single	191 (82.3%)
Married/cohabiting	35 (15.1%)
Divorced	6 (2.6%)
Age (years), mean ± SD	28.3 ± 9.5
Age at the time of the first psychiatric diagnosis (years), mean ± SD	21.31 ± 8.46
Age at ADHD diagnose (years), mean ± SD	24.67 ± 9.28
Age at first ADHD treatment (years), mean ± SD	24.34 ± 9.55
Employment status, *n* (%)	
Unemployed	46 (19.9%)
Student	91 (39.2%)
Manual worker	16 (6.9%)
Office worker	79 (34%)
Education (years), mean ± SD	14.34 ± 3.18
Referred for consultation by, *n* (%)	
Specialist	114 (49.13%)
Patient initiative	118 (50.86%)
Family history of psychiatric disorders, *n* (%)	118 (50.9%)
Family history of ADHD, *n* (%)	27 (11.6%)
Adult ADHD subtype, *n* (%)	
Inattentive subtype	79 (53%)
Combined subtype	70 (47%)
DIVA 2.0, mean (SD)	
Inattentive symptoms in childhood	5.54 ± 2.67
Hyperactivity symptoms in childhood	3.98 ± 2.85
Inattentive symptoms in adulthood	6.23 ± 2.14
Hyperactivity symptoms in adulthood	4.29 ± 2.49
Baseline ADHD-RS score, mean ± SD	35.57 ± 8.29
Lifetime psychiatric diagnoses, *n* (%)	
Learning disorders	29 (12.9%)
Anxiety disorders	44 (19%)
Bipolar disorders	30 (12.9%)
Major depressive disorder	64 (27.6%)
Personality disorders	26 (11.2%)
Eating disorder	13 (5.6%)
Obsessive- compulsive disorders	7 (3%)
Substance use disorder	41(17.7%)
Intellectual disability	6 (2.6%)
Autism spectrum disorders	16 (6.9%)
Impulsivity disorders	3 (1.3%)
Post-traumatic stress disorder	2 (0.9%)
Psychotic disorders	5 (2.2%)
Areas of functional impairment, *n* (%)	
Social functioning	130 (59.9%)
Relational functioning	125 (57.6%)
Academic functioning	160 (73.4%)
Occupational functioning *(26 non-evaluable)*	117 (56.8%)
Related symptoms, *n* (%)	
Mood swings	115 (49.6%)
Anger outbursts	86 (37.1%)
Low self-esteem	122 (52.6%)
Low tolerance for frustrations	103 (44.4%)
Delayed sleep onset	100 (43.1%)
Confirmed ADHD diagnosis, *n* (%)	149 (64.2%)

*Note:* Demographic and clinical characteristics of the sample (n = 232). Data are presented as mean ± SD for continuous variables and n (%) for categorical variables. ADHD-RS = ADHD Rating Scale; DIVA 2.0 = Diagnostic Interview for ADHD in adults, version 2.0.

Overall, the majority of patients were male (57,8%), with a mean age of 28,3 years, and 49,1% were referred by other healthcare professionals (psychiatrists, psychologists) rather than self-referred. The mean years of education were 14.3; 82.3% were single, 19.9% were unemployed, and 39.2% were students. A family history of ADHD in first-degree relatives was present in 11.6% of cases. Sixteen patients did not have an available caregiver, so the DIVA was not administered; instead, the BAARS-IV was completed.

Among the total sample, 64.2% received a confirmed diagnosis of ADHD.

Table 2 shows the demographic and clinical features of the two subgroups (ADHD versus non-ADHD) compared according to χ2 tests or the Kruskal–Wallis H test.

**Table 2.** Sociodemographic and Clinical Features of the Two Subgroups (ADHD versus non-ADHD) Compared According to χ2 Tests or Kruskal-Wallis H Test Table 2. long description.

Characteristics	ADHD group *n* = 149 (64.2%)	Non-ADHD group *n* = 83 (35.8%)	*p*-value
Sex, *n* (%)			
Male	91 (61.1%)	43 (51.8%)	0.171
Female	58 (38.9%)	40 (48.2%)	
Marital status, *n* (%)			
Single	102 (83.6%)	50 (79.4%)	0.775
Married/cohabiting	17 (13.9%)	11 (17.5%)	
Separated	3 (2.5%)	2 (3.2%)	
**Age (years), mean ± SD**	**27.34 ± 9.02**	**30.12 ± 10.46**	**0.035**
Age at the time of the first psychiatric diagnosis (years), mean ± SD	21.26 ± 9013	21.43 ± 7041	0.905
Age at ADHD diagnose (years), mean ± SD	24.73 ± 9.08	–	–
Age at first ADHD treatment (years), mean ± SD	24.50 ± 8.98	–	–
Employment status, *n* (%)			
Unemployed	24 (18.3%)	15 (20.5%)	
Student	52 (39.7%)	30 (41.1%)	0.860
Manual worker	9 (6.9%)	3 (4.1%)	
Office worker	45 (34.4%)	25 (34.2%)	
**Educational level (years), mean ± SD**	**13.94 ± 3189**	**15.06 ± 3070**	**0.017**
**Referred by, *n* (%)**			
**Healthcare professional**	**82 (55%)**	**32 (38.6%)**	**0.016**
**Self-referred**	**67 (45%)**	**51 (61.4%)**	
Family history of psychiatric disorders, *n* (%)	82 (58.6%)	36 (46.8%)	0.094
Family history of ADHD, *n* (%)	21 (15%)	6 (7.8%)	0.124
Adult ADHD subtype, *n* (%)			
Inattentive subtype	78 (52.7%)	–	–
Combined subtype	70 (47.3%)		
**DIVA 2.0, mean ± SD**			
**Inattentive symptoms in childhood**	**7.25 ± 1194**	**2.92 ± 2122**	**< 0.001**
**Hyperactivity symptoms in childhood**	**4.81 ± 2799**	**2.71 ± 2452**	**< 0.001**
**Inattentive symptoms in adulthood**	**7.16 ± 1394**	**4.79 ± 2313**	**< 0.001**
**Hyperactivity symptoms in adulthood**	**4.84 ± 2455**	**3.44 ± 2316**	**< 0.001**
Baseline ADHD-RS score, mean ± SD	35.98 ± 8239	34.85 ± 8441	0.464
**Areas of functional impairment, *n* (%)**			
**Social functioning, *n* (%)**	**106 (71.6%)**	**31 (39.2%)**	**< 0.001**
**Relational functioning, *n* (%)**	**103 (69.6%)**	**29 (36.7%)**	**< 0.001**
**Academic functioning, *n* (%)**	**132 (89.2%)**	**32 (40.5%)**	**< 0.001**
**Occupational functioning, *n* (%)**	**95(71.4%)**	**22 (30.1%)**	**< 0.001**
Non evaluable, *n* (%)	**16 (10.8%)**	**10 (12.8%)**	
**Related symptoms, *n* (%)**			
**Mood swings**	**95 (65.5%)**	**20 (25.3%)**	**< 0.001**
**Anger outbursts**	**70 (48.6%)**	**16 (20.5%)**	**< 0.001**
**Low self-esteem**	**88 (61.5%)**	**34 (43.6%)**	**0.010**
**Low tolerance to frustrations**	**83 (58%)**	**20 (26%)**	**< 0.001**
**Delayed sleep onset**	**87 (60%)**	**13 (16.7%)**	**< 0.001**
Lifetime psychiatric diagnoses, *n* (%)			
**Learning disorders**	**26 (17.9%)**	**3 (3.8%)**	**0.003**
**Anxiety disorders**	**19 (12.8%)**	**25 (30.1%)**	**0.001**
Bipolar disorders	21(14.1%)	9 (10.8%)	0.479
Depressive major disorders	40 (26.8%)	24 (28.9%)	0.735
Personality disorders	13 (8.7%)	13 (15.7%)	0.108
Eating disorders	7 (4.7%)	6 (7.2%)	0.422
Obsessive- compulsive disorders	3 (2.0%)	4 (4.8%)	0.231
Substance use disorders	30 (20.1%)	10 (12%)	0.118
Intellectual disability	2 (1.3%)	4 (4.8%)	0.110
Autism	12 (8.1%)	4 (4.8%)	0.351
Impulsivity disorders	1 (0.67%)	2 (2.40%)	–
Post traumatic stress disorder	2 (1.34%)	0 (0%)	–
Psychotic disorders	3 (2.0%)	2 (2.4%)	0.842

*Note:* Sociodemographic and clinical features of ADHD and non-ADHD subgroups. Continuous variables are presented as mean ± SD; categorical variables as n (%). ADHD-RS = ADHD Rating Scale; DIVA 2.0 = Diagnostic Interview for ADHD in adults, version 2.0. Group comparisons were performed using χ^2^ tests for categorical variables and Kruskal-Wallis H test for continuous variables.

The bold represents results with statistical significance (*p* < 0.05).

Patients with a confirmed diagnosis of ADHD showed significantly younger age (27.3 versus 30.1, *p* = 0.035) and significantly lower educational level (13.9 versus 15.1, *p* = 0.017) than those without an ADHD diagnosis. Furthermore, the confirmed ADHD group was more frequently referred by a healthcare professional-rather than self-referred - compared to the non-ADHD group (55% versus 38.6%, *p* = 0.016).

In terms of psychiatric comorbidities, the ADHD group (compared to the non-ADHD group) showed more frequent comorbidity with specific learning disorders (17.9% versus 3.8%, *p* = 0.003) and less frequent comorbidity with anxiety disorders (12.8% versus 30.1%, *p* = 0.001).

Those who received an ADHD diagnosis exhibited significantly higher prevalence of mood swings (65.5% versus 24%, *p* < 0.001), anger outbursts (48.6% versus 19.3%, *p* < 0.001), delayed sleep onset (60% versus 15.6%, *p* < 0.001), low tolerance to frustration (58% versus 24.1%, *p* < 0.001), and low self-esteem (61.5% versus 41%, *p* = 0.001).

Moreover, the ADHD group showed higher rates of impairment in occupational (71,4% versus 30,1%, *p* < 0.001), academic (88.6% versus 38.5%, *p* < 0.001), relational (69.1% versus 35%, *p* < 0.001), and social functioning (71.1% versus 37.3%, *p* < 0.001).

Regarding psychometric testing, DIVA interview scores were significantly higher among patients diagnosed with ADHD, both for childhood symptoms (inattention: 7.25 versus 2.92, *p* < 0.001; hyperactivity/impulsivity: 4.81 versus 2.71, *p* < 0.001) and for adult symptoms (inattention: 7.16 versus 4.79, *p* < 0.001; hyperactivity/impulsivity: 4.84 versus 3.44, *p* < 0.001).

No significant differences emerged between the ADHD and non-ADHD groups in ADHD-RS total scores (35.98 versus 34.85, *p* = 0.464).

These variables with a statistically significant difference (age, educational level, referral source, comorbidity with learning disorders and anxiety disorders, mood swings, anger outbursts, delayed sleep onset, low tolerance to frustration, low self-esteem, impairment in occupational, academic, relational and social functioning, DIVA interview scores in childhood and adulthood) were then subjected to binary logistic regression. After controlling for the other variables included in the model (39 missing cases), the following factors remained significantly associated with an ADHD diagnosis: referral source (professional referral versus self-referral) (*p* = 0.036, OR 3.294, 95% CI 1.079–10.053), academic impairment (*p* < 0.001, OR 9.950, 95% CI 3.338–29.219), DIVA inattention scores in childhood (*p* < 0.001, OR 3.860, 95% CI 1.739–8.569), mood swings (*p* < 0.001, OR 5.174, 95% CI 2.041–13.116)and delayed sleep onset (*p* < 0.001, OR 4.561, 95% CI 1.867–11.145).

The results of the binary logistic regression model are described in Table 3.

**Table 3.** The Relationship between Potential Explanatory Variables and ADHD Diagnosis: Results from the Binary Logistic Regression Analysis (*n* = 193) Table 3. long description.

	*B*	SE	Wald	*p*-value	OR	95%CI
Referral source	1.192	0.569	4.384	0.036	3.294	1.079–10.053
Childhood DIVA inattention score	1.351	0.407	11.017	<0.001	3.860	1.739–8.569
Mood swings	1.644	0.475	11.992	<0.001	5.174	2.041–13.116
Delayed sleep onset	1.518	0.456	11.086	<0.001	4.561	1.867–11.145
Academic impairment	2.298	0.550	17.476	<0.001	9.950	3.338–29.219
Constant	−2.998	0.999	9.008	0.003	0.050	

*Note:* Binary logistic regression predicting ADHD diagnosis (n = 193). *B* = regression coefficient; SE = standard error; *Wald* = Wald χ^2^; OR = odds ratio; 95% CI = 95% confidence interval. OR > 1 indicates increased odds of ADHD. Total variables initially entered in the model: n = 18. Significance set at *p* < 0.05.

No evidence of problematic multicollinearity was observed among the variables included in the regression model (VIF range 1.2–2.0).

**Figure 1.**
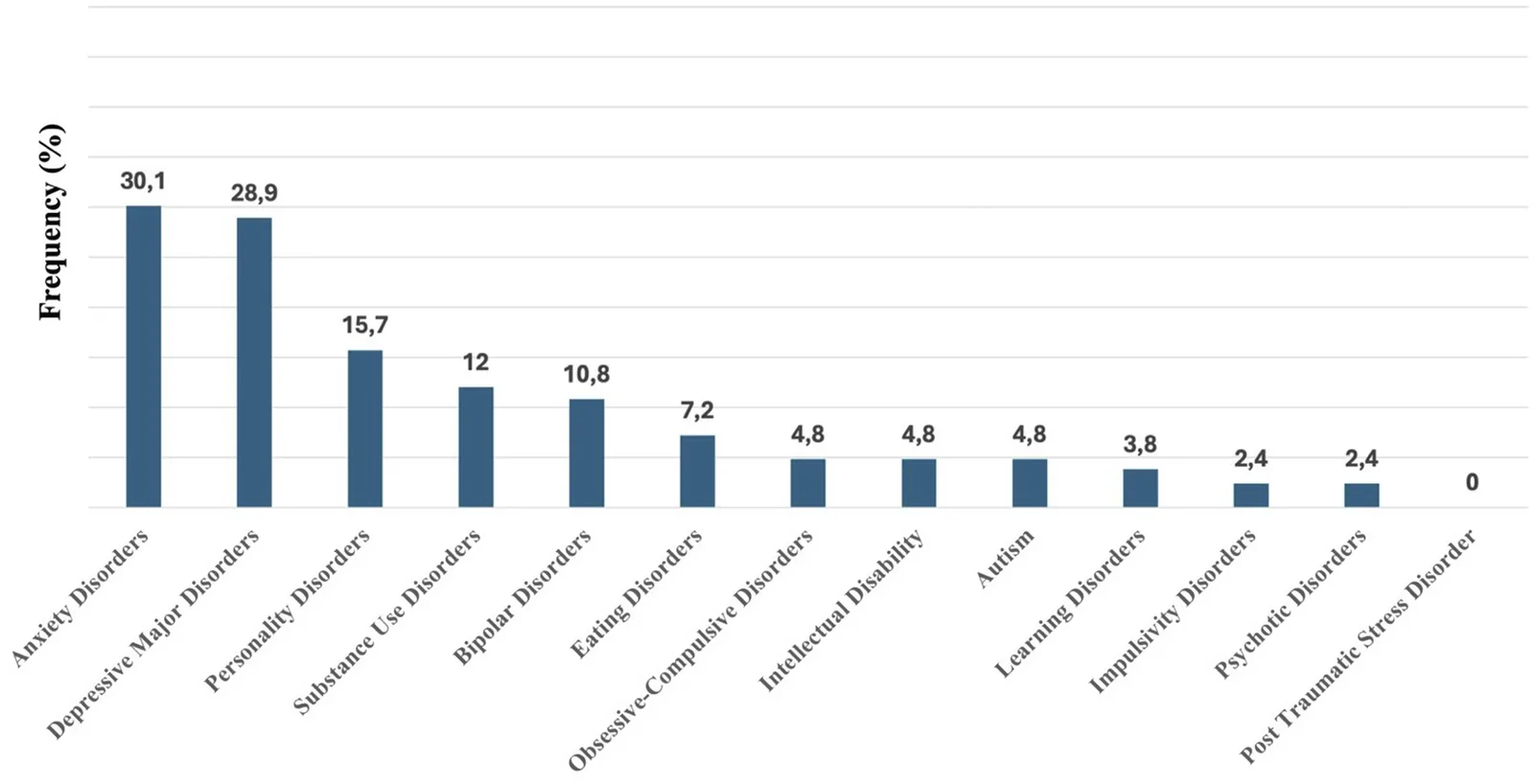
Psychiatric diagnoses in patients without ADHD. Figure 1. long description.

Finally, among participants who did not meet criteria for ADHD, several alternative psychiatric diagnoses were identified, as shown in Figure 1. The most frequent diagnoses were anxiety disorders (30.1%) and depressive disorders (28.9%), followed by personality disorders (15.7%) and substance use disorder (12%). Bipolar disorders were also present in 10.8% of these patients.

## Discussion

In this study, we aimed to explore the sociodemographic and clinical profiles of adults seeking ADHD evaluation, identify factors associated with a confirmed ADHD diagnosis, and assess the alternative diagnoses among those who did not meet diagnostic criteria for ADHD. It is important to note that several variables included in the analyses (e.g., DIVA scores and functional impairment) are conceptually embedded within the DSM-5 diagnostic framework. Therefore, the present findings should be interpreted as reflecting the relative contribution of different components within a clinically integrated diagnostic process, rather than as evidence of independent predictive validity.

Our sample consisted predominantly of young adults, with a mean age of 28.3 years, which aligns with the typical age range for seeking ADHD evaluation in adulthood.^22^ Our study cohort exhibited a marginally higher proportion of males (57.8%), while diagnostic rates did not differ significantly between genders, supporting evidence that the historical gender gap is narrowing over time.^29^ Of the patients evaluated for suspected ADHD, less than half (49.1%) were referred by another mental health specialist, an interesting finding that highlights the significant impact of self-referral and which appears to be higher compared to the Icelandic study, where only about one in four patients were referred by another professional.^22^ The fact that a larger proportion of our patients are referred by other specialists, compared with Iceland, may be related to the role of our clinic as the regional coordinating center for ADHD, which naturally receives referrals from other mental health professionals. In Italy, specialized ADHD services are limited,^30^ and patients often first access non-specialized psychiatric services; after an initial screening, they are subsequently referred to our center for a comprehensive assessment.

Overall, 64.2% of the patients received an ADHD diagnosis, in line with Suzuki et al.^20^ and Ómarsdóttir et al.^22^ although this rate is lower than that reported in another study,^21^ where 86.2% of individuals were diagnosed with ADHD, it is important to consider that their sample consisted exclusively of patients referred by other specialists—a more selectively pre-screened population.

Consistent with this interpretation, in our study, patients referred by healthcare professionals were also more likely to receive a confirmed ADHD diagnosis compared to those who self-referred, reflecting the importance of clinical judgment in identifying ADHD, as self-reported symptoms alone may not provide enough diagnostic clarity. Furthermore, professional referral may also help limit overdiagnosis or misdiagnosis by ensuring an initial screening step and, in turn, reducing waiting lists for adult ADHD assessments, which in Italy are conducted exclusively by specialized centers and are consequently very long.

Younger age was significantly associated with ADHD diagnosis in the univariate analyses, in line with previous studies employing similar methodologies.^22^ A possible explanation for the younger age observed in the ADHD group may relate to the developmental stage at which individuals typically seek evaluation: academic transitions and early professional years are challenging periods in which executive demands markedly increase, making difficulties in attention, organization, and self-regulation more apparent.^31^ However, the association between age and ADHD diagnosis did not remain significant in the multivariable model, suggesting that age may not represent an independent factor once core clinical and functional variables are taken into account. Furthermore, this finding may also partly reflect ascertainment processes, as younger adults may be more exposed and responsive to increasing ADHD-related content and awareness through digital and social media platforms.^32^ Patients with a confirmed diagnosis exhibited more frequent impairments in academic, occupational, relational, and social domains. This is consistent with findings from a similar study, which showed that, despite comparable levels of self-reported symptoms, individuals who received an ADHD diagnosis—rather than another diagnosis-displayed greater functional impairment, further underscoring the limited clinical specificity of self-perceived symptom severity in ADHD.^21^ This aligns with the well-established understanding that ADHD leads to pervasive functional difficulties across multiple life domains.^33^

However, in the logistic regression model, only academic impairment remained significantly associated with ADHD diagnosis. These findings suggest that difficulties in this functional area may serve as key clinical markers for ADHD, more specifically than impairments in other functionings, which typically play a comparatively greater role in other psychiatric conditions, as highlighted by previous studies.^34^ The fact that relational and social impairments were also reported more frequently in the ADHD group, yet did not remain significant in the multivariate analysis, may also indicate that they are likely influenced by additional factors—such as comorbid conditions or contextual variables—rather than being directly attributable to the core symptoms of ADHD. A similar consideration applies to occupational impairment. Although it was more frequently observed in the ADHD group in univariate analyses, it did not retain statistical significance in the adjusted model. This finding should be interpreted with caution, as occupational functioning was not evaluable in a considerable proportion of the sample (*n* = 26), largely due to the relatively young age of many participants, who were not yet engaged in stable employment while still studying. This represents a limitation of the study and may have reduced the ability to detect a clear independent association between occupational impairment and ADHD diagnosis.

The analysis of associated symptoms revealed that mood swings, anger outbursts, low self-esteem, low frustration tolerance, and delayed sleep onset were significantly more prevalent in the ADHD group. These findings are consistent with the literature, which increasingly recognizes emotional dysregulation as a central feature of ADHD, particularly in adults.^35^ Moreover, sleep disturbances are frequently reported in this population, most commonly manifesting as delayed sleep phase syndrome.^36^

However, only mood swings and delayed sleep onset remained significant predictors in the logistic regression model. Mood swings, in particular, are well-documented in ADHD and are thought to result from challenges in regulating emotional responses and impulsive behaviors.^37^ It is important to note that mood swings, as assessed in this study, refer to rapid and reactive emotional fluctuations rather than the episodic mood changes characteristic of bipolar disorder, although some degree of overlap with other psychiatric conditions cannot be entirely excluded. Sleep disturbances, such as delayed sleep onset, are also frequently reported in individuals with ADHD and may be related to heightened arousal or difficulties in managing attention, which interfere with the ability to wind down at night.^38^ Interestingly, other symptoms such as low self-esteem and low frustration tolerance were reported more frequently in the ADHD group but did not emerge as significant predictors in the logistic regression model. This suggests that, although these traits are commonly observed in individuals with ADHD, they may reflect less intrinsic aspects of the adult ADHD phenotype, being less diagnostically specific and may instead represent secondary consequences of age, functional impairment, or manifestations of comorbid conditions. For example, the literature shows that self-esteem and anger outbursts tend to improve with age.^39^ Consequently, the association observed between self-esteem and an ADHD diagnosis may be partly mediated by the age of individuals with ADHD, who in our sample were on average about three years younger than those who did not receive the diagnosis.

Regarding the DIVA scores—which assess childhood and adult inattention as well as childhood and adult hyperactivity/impulsivity—although all domains were associated with the ADHD diagnosis in the first-level statistical analyses, only childhood inattention retained a significant association with the clinician’s final diagnostic judgment in the multivariable model. Given that DIVA scores reflect DSM-5 diagnostic criteria, these findings should not be interpreted as identifying independent predictors, but rather as indicating which components of the diagnostic framework were most strongly aligned with the clinician’s final judgment. In this context, childhood inattention emerged as the most salient and specific component within the diagnostic process. This may reflect both the requirement of symptom onset in childhood according to DSM-5 criteria and the greater specificity of early inattentive symptoms, whereas adult symptoms may overlap with other psychiatric conditions such as anxiety, mood or substance use disorders.^40^ This further underscores the central role of early symptoms in the diagnostic process and supports the view that inattention may constitute the core feature of ADHD. Indeed, a purely hyperactive – impulsive presentation is extremely rare,^3^ whereas inattention is the most predominant feature in adulthood.^41^ Consistently, neither hyperactivity nor impulsivity—whether in childhood or adulthood—remained strongly associated with the diagnosis in the regression model, indicating that although these symptoms are primary, they may be less specific than inattention. This highlights the importance of obtaining a detailed developmental history during adult ADHD evaluations, as early manifestations of inattention appear to play a particularly salient role within the diagnostic process, although they may be difficult to reconstruct retrospectively.

While the DIVA—an interviewer-administered instrument—showed scores that were clearly associated with the ADHD diagnosis, it is noteworthy that the results of the ADHD Rating Scale (ADHD-RS), a self-report questionnaire, appeared comparable between the two subgroups. This finding further supports the limited clinical specificity of self-report measures in distinguishing adult ADHD from other psychiatric mimicking conditions within an assessment-seeking population, where high symptom endorsement is common to both groups, as already reported in the literature.^8^

Finally, individuals who did not receive an ADHD diagnosis were most frequently diagnosed with anxiety disorders and major depressive disorder, followed by personality disorders, substance use disorder, and bipolar disorders. These conditions therefore appear to be the ones that most commonly “mimic” ADHD and should be carefully assessed during the diagnostic evaluation. These findings are consistent with the limited evidence available in the literature, which has identified anxiety and mood disorders as the most frequent alternative diagnoses among adults evaluated for suspected ADHD.^8^ ^,^^22^ ^,^^23^ It is important to note, however, that these same disorders also emerged frequently as comorbidities within the ADHD group, and second-level statistical analyses did not reveal significant differences between the two subgroups (although first-level analyses showed an association between anxiety disorders and the non-ADHD group, and an association between learning disorders and the ADHD group). Altogether, these findings suggest that the presence of specific psychiatric disorders does not substantially influence whether individuals ultimately receive an ADHD diagnosis. Nonetheless, comorbidities remain highly relevant clinically, as their management typically represents the first therapeutic priority.^42^

### Strengths and limitations

This study has several strengths, including the use of a large Italian clinical cohort of assessment-seeking adults, a population still underrepresented in the literature. ADHD diagnoses were established through a comprehensive, gold-standard protocol incorporating the DIVA interview with collateral information, and a multidomain approach was adopted to capture core symptoms alongside functional impairment, emotional dysregulation, and sleep disturbances, providing a nuanced characterization of adult ADHD. However, some limitations should be acknowledged. The cross-sectional design precludes causal inferences, the single-center setting may limit generalizability, and the retrospective assessment of childhood symptoms is subject to recall bias, although partially mitigated by the inclusion of caregiver reports. No formal adjustment for multiple comparisons was applied in the univariate analyses. Given the number of statistical tests performed, the possibility of type I error (false positive findings) cannot be excluded, and results should therefore be interpreted with caution. Missing data were handled using a complete-case approach; although no obvious pattern suggesting systematic association with the outcome was observed descriptively, residual bias related to missingness cannot be excluded. Additionally, the relatively low events-per-variable ratio may have limited the stability of the regression estimates. Furthermore, although the overall pattern of results was clinically coherent, some confidence intervals were relatively wide, likely reflecting the number of predictors relative to the sample size, and findings should therefore be interpreted with caution.

## Conclusion

In conclusion, our study outlines a multifactorial profile underlying the diagnosis of ADHD, shaped by an interplay of clinical factors. This study provides the first systematic evidence from an Italian cohort, uniquely integrating DIVA-based confirmation with a multi-domain analysis of functional, emotional, and sleep-related predictors. Structured clinical tools, functional characteristics, and the referral context emerge as central contributors to diagnostic accuracy, whereas certain self-reported or more general factors appear to be less informative. Our findings indicate that adult ADHD is most strongly associated with specific functional impairments in academic and occupational domains, as well as with emotional-regulation difficulties such as mood lability and sleep disturbances. These features, together with childhood inattention—which remains a key historical indicator—appear to constitute the most reliable markers of the disorder, underscoring the importance of incorporating developmental trajectories into the diagnostic process. Given the limited clinical specificity of subjective reports and the paucity of evidence regarding the usefulness and specificity of neurocognitive test batteries,^43^ in recent years a neurocognitive testing battery has been incorporated into our assessment protocol for suspected ADHD (with data collection currently ongoing), and it may constitute a valuable component of the diagnostic process.

The results of the present study carry practical implications for the early identification and targeted management of adults presenting with suspected ADHD. Further research involving larger samples and conducted across diverse clinical settings will be essential to validate and extend these findings.

## Long descriptions

### Table 1. Long description

The table presents data for a sample of n = 232.

* Sex: Male 134 (57.8 percent), Female 98 (42.2 percent).

* Marital status: Single 191 (82.3 percent), Married or cohabiting 35 (15.1 percent), Divorced 6 (2.6 percent).

* Age: 28.3 plus or minus 9.5 years.

* Clinical Timelines (mean plus or minus S D): First psychiatric diagnosis at 21.31 plus or minus 8.46 years; A D H D diagnosis at 24.67 plus or minus 9.28 years; First A D H D treatment at 24.34 plus or minus 9.55 years.

* Employment: Unemployed 46 (19.9 percent), Student 91 (39.2 percent), Manual worker 16 (6.9 percent), Office worker 79 (34 percent).

* Education: 14.34 plus or minus 3.18 years.

* Referral: Specialist 114 (49.13 percent), Patient initiative 118 (50.86 percent).

* Family History: Psychiatric disorders 118 (50.9 percent), A D H D 27 (11.6 percent).

* Adult A D H D Subtype: Inattentive 79 (53 percent), Combined 70 (47 percent).

* D I V A 2.0 Scores (mean plus or minus S D): Childhood inattentive 5.54 plus or minus 2.67, Childhood hyperactivity 3.98 plus or minus 2.85, Adulthood inattentive 6.23 plus or minus 2.14, Adulthood hyperactivity 4.29 plus or minus 2.49.

* Baseline A D H D R S score: 35.57 plus or minus 8.29.

* Lifetime Psychiatric Diagnoses: Major depressive disorder 64 (27.6 percent), Anxiety disorders 44 (19 percent), Substance use disorder 41 (17.7 percent), Bipolar disorders 30 (12.9 percent), Learning disorders 29 (12.9 percent), Personality disorders 26 (11.2 percent), Autism spectrum disorders 16 (6.9 percent), Eating disorder 13 (5.6 percent), Obsessive-compulsive disorders 7 (3 percent), Intellectual disability 6 (2.6 percent), Psychotic disorders 5 (2.2 percent), Impulsivity disorders 3 (1.3 percent), Post-traumatic stress disorder 2 (0.9 percent).

* Functional Impairment: Academic 160 (73.4 percent), Social 130 (59.9 percent), Relational 125 (57.6 percent), Occupational 117 (56.8 percent).

* Related Symptoms: Low self-esteem 122 (52.6 percent), Mood swings 115 (49.6 percent), Low tolerance for frustrations 103 (44.4 percent), Delayed sleep onset 100 (43.1 percent), Anger outbursts 86 (37.1 percent).

* Confirmed A D H D diagnosis: 149 (64.2 percent).

Navigate back to Table 1..

### Table 2. Long description

The table compares an A D H D group (n = 149) and a Non-A D H D group (n = 83).

Key significant differences (p < 0.05) include:

* Age: A D H D group mean 27.34 plus or minus 9.02 years vs Non-A D H D 30.12 plus or minus 10.46 (p = 0.035).

* Educational level: A D H D group 13.94 plus or minus 3189 vs Non-A D H D 15.06 plus or minus 3070 (p = 0.017).

* Referral source: Self-referral was higher in the Non-A D H D group (61.4 percent) compared to the A D H D group (45 percent) (p = 0.016).

* D I V A 2.0 scores: The A D H D group scored significantly higher in all categories (Inattentive and Hyperactivity symptoms in both childhood and adulthood) with p < 0.001.

* Functional impairment: The A D H D group showed significantly higher impairment in Social (71.6 percent), Relational (69.6 percent), Academic (89.2 percent), and Occupational (71.4 percent) functioning (all p < 0.001).

* Related symptoms: A D H D patients reported significantly higher rates of mood swings (65.5 percent), anger outbursts (48.6 percent), low tolerance to frustrations (58 percent), and delayed sleep onset (60 percent) (all p < 0.001).

* Lifetime psychiatric diagnoses: Learning disorders were higher in the A D H D group (17.9 percent vs 3.8 percent, p = 0.003), while Anxiety disorders were higher in the Non-A D H D group (30.1 percent vs 12.8 percent, p = 0.001).

Non-significant factors (p > 0.05) included sex, marital status, employment status, family history of psychiatric disorders, and baseline A D H D-R S scores.

Navigate back to Table 2..

### Table 3. Long description

The table presents data for 193 participants across seven columns: Variable, B, S E, Wald, p-value, O R, and 95% C I.

* Referral source: B 1.192, S E 0.569, Wald 4.384, p-value 0.036, O R 3.294, 95% C I 1.079–10.053.

* Childhood D I V A inattention score: B 1.351, S E 0.407, Wald 11.017, p-value less than 0.001, O R 3.860, 95% C I 1.739–8.569.

* Mood swings: B 1.644, S E 0.475, Wald 11.992, p-value less than 0.001, O R 5.174, 95% C I 2.041–13.116.

* Delayed sleep onset: B 1.518, S E 0.456, Wald 11.086, p-value less than 0.001, O R 4.561, 95% C I 1.867–11.145.

* Academic impairment: B 2.298, S E 0.550, Wald 17.476, p-value less than 0.001, O R 9.950, 95% C I 3.338–29.219.

* Constant: B minus 2.998, S E 0.999, Wald 9.008, p-value 0.003, O R 0.050.

Note: B is the regression coefficient, S E is standard error, Wald is Wald chi-squared, O R is odds ratio, and 95% C I is the 95% confidence interval.

Navigate back to Table 3..

### Figure 1. Long description

The y-axis is labeled Frequency percent and ranges from 0 to 35 with horizontal grid lines. The x-axis lists 13 psychiatric categories. The data shows a downward trend from left to right.

* Anxiety Disorders: 30,1 percent.

* Depressive Major Disorders: 28,9 percent.

* Personality Disorders: 15,7 percent.

* Substance Use Disorders: 12 percent.

* Bipolar Disorders: 10,8 percent.

* Eating Disorders: 7,2 percent.

* Obsessive-Compulsive Disorders: 4,8 percent.

* Intellectual Disability: 4,8 percent.

* Autism: 4,8 percent.

* Learning Disorders: 3,8 percent.

* Impulsivity Disorders: 2,4 percent.

* Psychotic Disorders: 2,4 percent.

* Post Traumatic Stress Disorder: 0 percent.

Navigate back to Figure 1..

## References

[1] Volkow ND, Swanson JM. Clinical practice: adult attention deficit-hyperactivity disorder. N Engl J Med. 2013;369(20):1935–1944. doi:10.1056/NEJMcp1212625. PMC4827421 24224626

[2] Posner J, Polanczyk GV, Sonuga-Barke E. Attention-deficit hyperactivity disorder. Lancet. 2020;395(10222):450–462. doi:10.1016/S0140-6736(19)33004-1. PMC7880081 31982036

[3] Bogdańska-Chomczyk E, Majewski MK, Kozłowska A. ADHD in adulthood: clinical presentation, comorbidities, and treatment perspectives. Int J Mol Sci. 2025;26(22):11020. doi:10.3390/ijms262211020. PMC12652008 41303501

[4] Cortese S, Bellgrove MA, Brikell I, et al. Attention-deficit/hyperactivity disorder (ADHD) in adults: evidence base, uncertainties and controversies. World Psychiatry. 2025;24(3):347–371. doi:10.1002/wps.21374. PMC12434367 40948064

[5] The Ohio State University Wexner Medical Center. Survey finds 25 percent of adults suspect they have undiagnosed ADHD. Published 2024, accessed 30 October 2025. https://wexnermedical.osu.edu/mediaroom/pressreleaselisting/survey-finds-25-percent-of-adults-suspect-they-have-undiagnosed-adhd

[6] Italian Medicines Agency (AIFA). Analysis of data from the Italian methylphenidate registry (2019–2025). Rome, Italy: AIFA; 2025.

[7] Yeung A, Ng E, Abi-Jaoude E. TikTok and attention-deficit/hyperactivity disorder: a cross-sectional study of social media content quality. Can J Psychiatry. 2022;67(12):899–906. doi:10.1177/07067437221082854. PMC9659797 35196157

[8] Vizgaitis AL, Bottini S, Polizzi CP, Barden E, Krantweiss AR. Self-reported adult ADHD symptoms: evidence supporting cautious use in an assessment-seeking sample. J Atten Disord. 2023;27(10):1156–1166. doi:10.1177/10870547231172764. 37158158

[9] Van Voorhees EE, Hardy KK, Kollins SH. Reliability and validity of self- and other-ratings of symptoms of ADHD in adults. J Atten Disord. 2011;15(3):224–234. doi:10.1177/1087054709356163. PMC3556723 20424007

[10] Alexander SJ, Harrison AG. Cognitive responses to stress, depression, and anxiety and their relationship to ADHD symptoms in first year psychology students. J Atten Disord. 2013;17(1):29–37. doi:10.1177/1087054711413071. 21825110

[11] Huang H, Huang H, Spottswood M, Ghaemi N. Approach to evaluating and managing adult attention-deficit/hyperactivity disorder in primary care. Harv Rev Psychiatry. 2020;28(2):100–106. doi:10.1097/HRP.0000000000000248. 32134834

[12] Harrison AG, Nay S, Armstrong IT. Diagnostic accuracy of the Conners’ adult ADHD rating scale in a postsecondary population. J Atten Disord. 2019;23(14):1829–1837. doi:10.1177/1087054715625299. 26794674

[13] McCann BS, Roy-Byrne P. Screening and diagnostic utility of self-report attention deficit hyperactivity disorder scales in adults. Compr Psychiatry. 2004;45(3):175–183. doi:10.1016/j.comppsych.2004.02.006. 15124147

[14] Kooij JJ, Boonstra MA, Swinkels SH, Bekker EM, de Noord I, Buitelaar JK. Reliability, validity, and utility of instruments for self-report and informant report concerning symptoms of ADHD in adult patients. J Atten Disord. 2008;11(4):445–458. doi:10.1177/1087054707299367. 18083961

[15] Lewandowski LJ, Lovett BJ, Codding RS, Gordon M. Symptoms of ADHD and academic concerns in college students with and without ADHD diagnoses. J Atten Disord. 2008;12(2):156–161. doi:10.1177/1087054707310882. 18192625

[16] Harrison AG, Beal AL, Armstrong IT. Predictive value of performance validity testing and symptom validity testing in psychoeducational assessment. Appl Neuropsychol Adult. 2023;30(3):315–329. doi:10.1080/23279095.2021.1943396. 34261385

[17] Binder LM. Deception and malingering. In: Crawford JR, Parker DM, McKinlay WW, eds. Handbook of Neuropsychological Assessment. Boston, MA: Springer; 1992:353–374. doi:10.1007/978-1-4899-0682-3_13.

[18] Canadian ADHD Resource Alliance (CADDRA). Canadian ADHD Practice Guidelines. Version 4.1. Toronto, ON, Canada: CADDRA; 2020.

[19] Oehrlein EM, Burcu M, Safer DJ, Zito JM. National trends in ADHD diagnosis and treatment: comparison of youth and adult office-based visits. Psychiatr Serv. 2016;67(9):964–969. doi:10.1176/appi.ps.201500269. 27181734

[20] Suzuki K, Niitsu T, Kato M, Iyo M. Reevaluation of ADHD diagnoses in referral patients: a medical chart review in an advanced psychiatric hospital in Japan. PCN Rep. 2024;3(4):e70024. doi:10.1002/pcn5.70024. PMC11472302 39403070

[21] Adamis D, West S, Singh J, et al. Functional impairment and quality of life in newly diagnosed adults attending a tertiary ADHD clinic in Ireland. Ir J Med Sci. 2024;193(5):2433–2441. doi:10.1007/s11845-024-03713-6. PMC11457577 38755511

[22] Ómarsdóttir BS, Kjartansdóttir SH, Magnússon P, Ólafsdóttir H, Sigurðsson JF. Adults referred to a national ADHD clinic in Iceland: clinical characteristics and follow-up status. Nord J Psychiatry. 2021;75(8):559–567. doi:10.1080/08039488.2021.1904287. 33840348

[23] Efron D, Bryson H, Lycett K, Sciberras E. Children referred for evaluation for ADHD: comorbidity profiles and characteristics associated with a positive diagnosis. Child Care Health Dev. 2016;42(5):718–724. doi:10.1111/cch.12364. 27273368

[24] First MB, Williams JBW, Karg RS, Spitzer RL. Structured Clinical Interview for DSM-5 Disorders: Clinician Version (SCID-5-CV). Arlington, VA: American Psychiatric Association; 2016.

[25] Kooij JJS, Francken MH, Bron TI. Diagnostic Interview for ADHD in Adults (3rd ed., DIVA-5th ed.). DIVA Foundation; 2019.

[26] Barkley RA. Barkley Adult ADHD Rating Scale–IV (BAARS-IV). New York, NY: Guilford Press; 2011.

[27] DuPaul GJ, Power TJ, Anastopoulos AD, Reid R. ADHD Rating Scale-IV: School Checklist (ADHD-RS-IV). APA PsycTests; New York, NY: Guilford Press; 1998.

[28] Skirrow C, Asherson P. Emotional lability, comorbidity and impairment in adults with attention-deficit hyperactivity disorder. J Affect Disord. 2013;147(1–3):80–86. doi:10.1016/j.jad.2012.10.011. 23218897

[29] Platania NM, Starreveld DEJ, Wynchank D, Beekman ATF, Kooij S. Bias by gender: exploring gender-based differences in the endorsement of ADHD symptoms and impairment among adult patients. Front Glob Womens Health. 2025;6:1549028. doi:10.3389/fgwh.2025.1549028. PMC11965619 40182228

[30] Zadra E, Giupponi G, Migliarese G, et al. Survey on centres and procedures for the diagnosis and treatment of adult ADHD in public services in Italy. Riv Psichiatr. 2020;55(6):355–365. doi:10.1708/3503.34894. 33349729

[31] Robb A, Findling RL. Challenges in the transition of care for adolescents with attention-deficit/hyperactivity disorder. Postgrad Med. 2013;125(4):131–140. doi:10.3810/pgm.2013.07.2685. 23933901

[32] Armstrong S, Osuch E, Wammes M, et al. Self-diagnosis in the age of social media: a pilot study of youth entering mental health treatment for mood and anxiety disorders. Acta Psychol. 2025;256:105015. doi:10.1016/j.actpsy.2025.105015. 40253960

[33] Kosheleff AR, Mason O, Jain R, Koch J, Rubin J. Functional impairments associated with ADHD in adulthood and the impact of pharmacological treatment. J Atten Disord. 2023;27(7):669–697. doi:10.1177/10870547231158572. PMC10173356 36876491

[34] Holst Y, Thorell LB. Functional impairments among adults with ADHD: a comparison with adults with other psychiatric disorders and links to executive deficits. Appl Neuropsychol Adult. 2020;27(3):243–255. doi:10.1080/23279095.2018.1532429. 30624968

[35] Beheshti A, Chavanon ML, Christiansen H. Emotion dysregulation in adults with attention deficit hyperactivity disorder: a meta-analysis. BMC Psychiatry. 2020;20(1):120. doi:10.1186/s12888-020-2442-7. PMC7069054 32164655

[36] van der Ham M, Bijlenga D, Böhmer M, Beekman ATF, Kooij S. Sleep problems in adults with ADHD: prevalences and their relationship with psychiatric comorbidity. J Atten Disord. 2024;28(13):1642–1652. doi:10.1177/10870547241284477. 39354860

[37] Lundervold AJ, Halmøy A, Nordby ES, Haavik J, Meza JI. Current and retrospective childhood ratings of emotional fluctuations in adults with ADHD. Front Psychol. 2020;11:571101. doi:10.3389/fpsyg.2020.571101. PMC7769943 33384637

[38] Valsecchi P, Nibbio G, Rosa J, Vita A. Adult ADHD and sleep disorders: prevalence, severity and predictors of sleep disorders in a sample of Italian psychiatric outpatients. Psychiatry Res. 2022;310:114447. doi:10.1016/j.psychres.2022.114447. 35192987

[39] Galambos NL, Barker ET, Krahn HJ. Depression, self-esteem, and anger in emerging adulthood: seven-year trajectories. Dev Psychol. 2006;42(2):350–365. doi:10.1037/0012-1649.42.2.350. 16569173

[40] Katzman MA, Bilkey TS, Chokka PR, Fallu A, Klassen LJ. Adult ADHD and comorbid disorders: clinical implications of a dimensional approach. BMC Psychiatry. 2017;17(1):302. doi:10.1186/s12888-017-1463-3. PMC5567978 28830387

[41] Bedawi RM, Al-Farsi Y, Mirza H, et al. Prevalence and clinical profile of adults with ADHD attending a tertiary care hospital for five years. Int J Environ Res Public Health. 2024;21(5):566. doi:10.3390/ijerph21050566. PMC11121453 38791781

[42] Kooij JJS, Bejerot S, Blackwell A, et al. European consensus statement on diagnosis and treatment of adult ADHD: the European network adult ADHD. BMC Psychiatry. 2010;10(1):67. doi:10.1186/1471-244X-10-67. PMC2942810 20815868

[43] Rosso G, Portaluppi C, Teobaldi E, Di Salvo G, Maina G. Assessing adult ADHD through objective neuropsychological measures: a critical overview. J Atten Disord. 2023;27(7):786–794. doi:10.1177/10870547231167564. 37039121

